# S-amlodipine plus chlorthalidone vs. S-amlodipine plus telmisartan in hypertensive patients unresponsive to amlodipine monotherapy: study protocol for a randomized controlled trial

**DOI:** 10.1186/s13063-018-2636-1

**Published:** 2018-06-20

**Authors:** Sang-Ho Jo, Sung-Ji Park, Eung Ju Kim, Soo-Joong Kim, Hyun-Jae Cho, Jong-Min Song, Jinho Shin, Jin Joo Park, Joon-Han Shin, Kyoo-Rok Han, Dong-Ju Choi

**Affiliations:** 1 Division of Cardiology, Hallym University Sacred Heart Hospital/Hallym University College of Medicine, 896, Pyeongchon-dong, Dongan-gu, Anyang-si, Gyeonggi-do 431-070 South Korea; 2 Division of Cardiology, Heart Vascular Stroke Institute, Samsung Medical Center/Sungkyunkwan University School of Medicine, #50 Irwon-dong, Gangnam-gu, Seoul, 135-710 South Korea; 3 Cardiovascular Center, Korea University Guro Hospital, Seoul, South Korea; 4 Division of Cardiology, KyungHee University Hospital, Seoul, South Korea; 5 Division of Cardiology, Seoul National University Hospital, Seoul, South Korea; 6 Division of Cardiology, University of Ulsan College of Medicine/Asan Medical Center, Seoul, South Korea; 7 Division of Cardiology, Hanyang University Hospital, Seoul, South Korea; 8 Division of Cardiology, Department of Internal Medicine, Seoul National University Bundang Hospital, Seongnam-si, Gyeonggi-do 463-707 South Korea; 9 Department of Cardiology, Ajou University School of Medicine, Suwon, South Korea; 10 Department of Internal Medicine, Kangdong Sacred Heart Hospital/Hallym University Medical Center, Seoul, South Korea

**Keywords:** Hypertension, Combination, Calcium channel blocker, Angiotensin receptor blocker

## Abstract

**Background:**

The efficacy of a combination of a calcium channel blocker (CCB) plus chlorthalidone (diuretic) versus a CCB plus an angiotensin receptor blocker (ARB) in patients not responding to CCB monotherapy has not been evaluated previously. We plan to compare the efficacy and safety of S-amlodipine (CCB) plus chlorthalidone versus S-amlodipine plus telmisartan (ARB) combinations among hypertension patients unresponsive to amlodipine monotherapy.

**Methods/design:**

This study is a prospective, randomized, double-blind, multicenter, parallel, non-inferiority phase 4 study. Hypertension patients who have been treated with amlodipine (5 mg) or S-amlodipine (2.5 mg) monotherapy for ≥2 weeks and whose mean diastolic blood pressure (DBP) is greater than 90 mmHg will be randomized to either S-amlodipine (2.5 mg) plus chlorthalidone (25 mg) or S-amlodipine (2.5 mg) plus telmisartan (40 mg) therapy. The primary efficacy endpoint is mean sitting DBP change after 12 weeks of treatment. The study objective is to prove the non-inferiority of the former combination (test drug) as compared to the latter one (control) with a non-inferiority margin of 3 mmHg in mean DBP change. The secondary endpoints are 6-week DBP change, 6- and 12-week sitting systolic BP (SBP) change, and the attainment of the target BP (SBP < 140 mmHg or DBP < 90 mmHg). Urine albumin, albumin/creatinine ratio (ACR), pulse wave velocity, central BP, 24-h ambulatory BP monitoring, and body fluid composition analysis will be performed at each hospital’s discretion. The sample size was estimated as 170 in total with 1:1 randomization.

**Discussion:**

This is the first study comparing the efficacy of a CCB plus chlorthalidone versus a CCB plus an ARB in patients who are not responding to CCB single therapy. The study result will help clinicians to choose between chlorthalidone and telmisartan in CCB-unresponsive patients.

**Trial registration:**

ClinicalTrials.gov, NCT03226340. Registered on 2 December 2015.

**Electronic supplementary material:**

The online version of this article (10.1186/s13063-018-2636-1) contains supplementary material, which is available to authorized users.

## Background

Hypertension is the most prevalent disease worldwide and a leading cause of cardiovascular mortality and morbidity [1]. Its treatment has proven prolonging survival and reducing ischemic heart disease and stroke [2].

Most patients with hypertension need to be treated with antihypertensive medication, and some require two or more antihypertensive drugs [3]. A Korean study reported that 46% of hypertension patients are treated with two or more drugs, 38% with two drugs, and 8% with three or more drugs [4]. Accordingly, it is important to determine the most appropriate combination of antihypertensive agents. However, clinical trials comparing each combination therapy are scarce.

In the representative large-scale “Avoiding Cardiovascular Events in Combination Therapy in Patients Living with Systolic Hypertension (ACCOMPLISH)” study, the efficacy of an angiotensin-converting enzyme inhibitor (ACEI) plus a calcium channel blocker (CCB) was compared with that of an ACEI plus a diuretic, and the study demonstrated the superiority of the former combination based on clinical outcomes, despite a similar blood pressure (BP) reduction in both groups [5].

Among possible two-drug combination regimens, the European Society of Cardiology (ESC) guideline on arterial hypertension recommends CCBs and diuretics as the central drugs for the combination [6]. The guideline suggests addition of ACEIs, angiotensin receptor blockers (ARBs), and diuretics to CCB monotherapy, and as add-on drugs to diuretics, ARBs, ACEIs, and CCBs are recommended. Considering the similarity between ACEIs and ARBs, we can assume the major three-combination regimens as ACEI/ARB + CCB, ACEI/ARB + diuretic, and CCB + diuretic.

Combination regimen comparisons other than that mentioned in the ACCOMPLISH study (ACEI/ARB + CCB versus ACEI/ARB + diuretic) are ([ACEI/ARB + CCB] versus [CCB + diuretic]) and ([ACEI/ARB + diuretic] versus [CCB + diuretic]). Our study deals with the former one.

Each drug of the CCB + diuretic combination (amlodipine/S-amlodipine and thiazide/chlorthalidone) has been extensively studied [7, 8], and the American and European guidelines on hypertension treatment recommend the combination of CCB + diuretic [9, 10]. This combination has demonstrated good efficacy and safety in some studies [11–13]. However, it is not as commonly used as the renin–angiotensin–aldosterone antagonist-based combination. The CCB + diuretic combination has only been compared with combinations, such as beta-blocker + diuretic [14] and ARB + diuretic [15], in large-scale clinical trials. A randomized clinical trial investigated the difference between CCB + diuretic and CCB + ARB combinations by comparing three regimens: benidipine + thiazide diuretic, benidipine + ARB, and benidipine + beta-blocker, and cardiovascular outcomes and BP-lowering effects with benidipine + thiazide and benidipine + ARB were similar [16]. In contrast to hydrochlorothiazide used in this reported trial, we have chosen chlorthalidone as the diuretic as it is associated with better clinical outcomes and BP-lowering efficacy and lower adverse effects than hydrochlorothiazide; however, this report is controversial [17, 18]. In fact, chlorthalidone is rarely used in Korea and hydrochlorothiazide is the most commonly used diuretic. Accordingly, there is a lack of data regarding single and combination therapies with chlorthalidone in Korea.

In this clinical trial, we plan to compare the efficacy and safety between S-amlodipine plus chlorthalidone (diuretic) combination as the test combination regimen and S-amlodipine plus telmisartan (ARB) as control in patients with hypertension who do not adequately respond to CCB monotherapy.

## Methods/design

### Overall design

This study is a prospective, randomized, double-blind, multicenter, parallel, non-inferiority phase 4 study to compare the efficacy and safety between S-amlodipine plus chlorthalidone (diuretic) and S-amlodipine plus telmisartan combination therapies in patients with hypertension who do not adequately respond to CCB monotherapy.

**Fig. 1:**
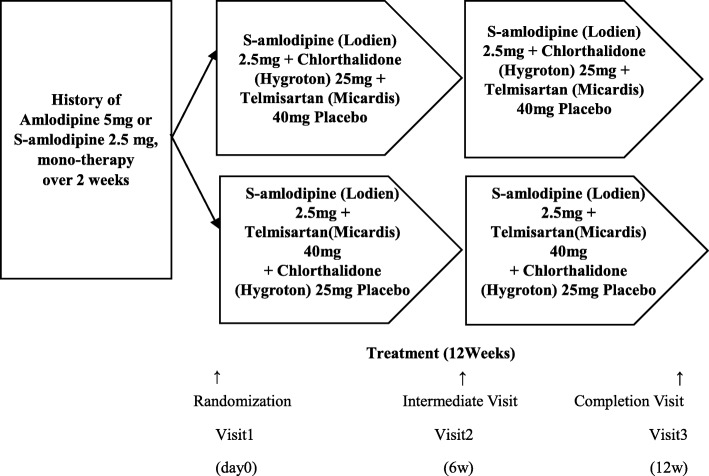
Overall scheme of the study

Ten tertiary university hospitals in Korea are to participate in this study. Patients with essential hypertension who have a medical history of treatment with amlodipine (5 mg) or S-amlodipine (2.5 mg) monotherapy for ≥2 weeks prior to screening and whose mean sitting diastolic BP (DBP) is >90 mmHg are considered as potential subjects for this study. Those who have completed the final compatibility evaluation will be randomly assigned to either the test group (S-amlodipine 2.5 mg + chlorthalidone 25 mg) or the control group (S-amlodipine 2.5 mg + telmisartan 40 mg) in a 1:1 ratio. The study subjects in both groups will receive an oral dose of the test or control drugs plus a matching placebo once daily for a total of 12 weeks and will be evaluated for safety and efficacy. They will visit the outpatient clinic three times, including screening during the study period (Fig. 1).

The trial is approved by the relevant institutional review board of each center and is registered at ClinicalTrials.gov (identifier no. NCT03226340).

**Fig. 2:**
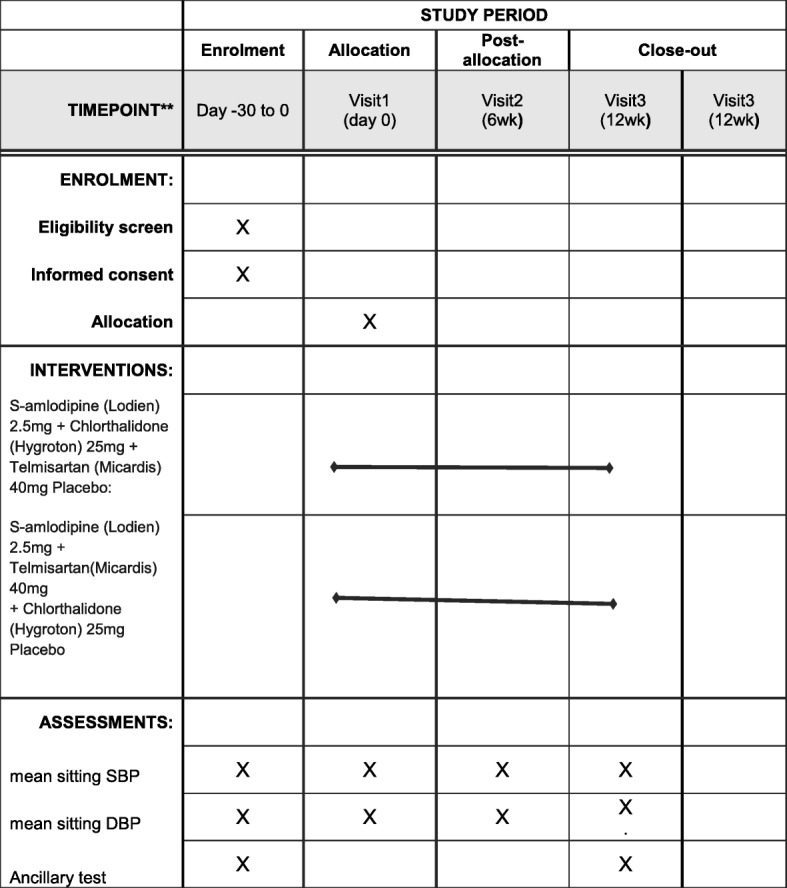
Standard Protocol Items: Recommendations for Interventional Trials (SPIRIT)

For a detailed overview, see the Standard Protocol Items: Recommendations for Interventional Trials (SPIRIT) figure (Fig. 2). The SPIRIT checklist is provided as Additional file 1.

### Study objective

The study aim is to demonstrate the non-inferiority (non-inferiority margin of 3 mmHg in DBP) of the S-amlodipine plus chlorthalidone combination therapy compared to the S-amlodipine plus telmisartan combination therapy in patients whose BP is not controlled with amlodipine monotherapy.

### Patients

#### Inclusion criteria

1. Male and female patients with essential hypertension, aged between 18 and 80 years.
2. Patients who are confirmed to be treated with amlodipine or S-amlodipine monotherapy for ≥2 weeks immediately prior to screening.
3. Patients whose mean sitting DBP is >90 mmHg, which is confirmed by triplicate measurements from the reference arm at screening.
4. Patients who have signed the informed consent by the study associate.

#### Exclusion criteria

1. Patients with a history of secondary hypertension and suspected secondary hypertension including coarctation of the aorta, primary hyperaldosteronism, renal artery stenosis, Cushing’s syndrome, pheochromocytoma, and polycystic kidney disease.
2. Patients with a mean systolic BP (SBP) ≥ 200 mmHg or DBP ≥ 110 mmHg at the screening visit.
3. Patients with ≥20 mmHg difference between the highest and the lowest sitting SBP or ≥10 mmHg difference between the highest and the lowest DBP, which is confirmed by triplicate measurements from the reference arm at screening.
4. Patients with uncontrolled diabetes (HbA1c ≥ 9.0%).
5. Patients who have been continuously taking other medicines, such as systemic steroids, thyroid hormones, oral contraceptives (except for menopausal hormone replacement therapy), psychiatric drugs, NSAIDs, sympathetic drugs, and immune suppressants, which have the potential to affect the BP.
6. Patients with symptomatic orthostatic hypotension.
7. Patients with a history of malignant tumors, including leukemia and lymphoma, within the past 5 years.
8. Patients with a history of autoimmune diseases, such as rheumatoid arthritis and systemic lupus erythematosus.
9. Patients with a history of hypersensitivity to S-amlodipine nicotinate or other drugs containing chlorthalidone or telmisartan.
10. Patients with clinically significant kidney and liver diseases, such as those on dialysis, liver cirrhosis, biliary obstruction, and hepatic failure, or those who show the following findings at screening visit:
  Alanine transaminase or aspartate transaminase level is at least three times higher than the normal upper limit.
  Total bilirubin level is more than twice the normal upper limit.
  Blood urea nitrogen level is more than twice the normal upper limit.
  Alkaline phosphatase level is more than twice the normal upper limit.
  Creatinine clearance level is less than 10 mL/min.
11. Patients with a history of the following diseases in the past 6 months, which are determined to be clinically significant by the investigator:
  Heart failure (NYHA classes III and IV), ischemic heart diseases (coronary artery diseases, such as angina pectoris and myocardial infarction), peripheral vascular diseases, hemodynamically significant valve stenosis, and arrhythmia.
  Severe cerebrovascular events, including stroke, cerebral infarction, and cerebral hemorrhage.
12. Patients with shock.
13. Patients with a history of alcohol or drug abuse.
14. Patients with potential pregnancy or who are breastfeeding.
15. Patients who are judged by the investigator to be inadequate to participate in the clinical study both legally and psychologically.
16. Patients who have participated in clinical studies with other investigational drug products within 4 weeks prior to screening.

### Study drugs

The test drugs are a combination of Lodien tablet, 2.5 mg (S-amlodipine 2.5 mg, Hanlim Pharmaceutical Co., Ltd.) plus Hygroton tablet, 25 mg (chlorthalidone 25 mg, Hanlim Pharmaceutical Co., Ltd.). The control drugs are a combination of Lodien tablet, 2.5 mg plus Micardis tablet, 40 mg (telmisartan 40 mg, Boehringer Ingelheim Korea Co., Ltd.). The patients are recommended to take both the tablets (test or control drugs) and matching placebo (totally three tablets) at the same time once a day for 12 weeks (Fig. 1).

### Endpoints

The primary endpoint is to demonstrate the non-inferiority of the S-amlodipine plus chlorthalidone combination therapy compared to the S-amlodipine plus telmisartan combination therapy based on the difference in sitting DBP after 12 weeks of treatment. The secondary endpoints are mean sitting DBP change at 6 weeks, 6- and 12-week sitting SBP change, and the attainment of the target BP (SBP < 140 mmHg or DBP < 90 mmHg) at 12 weeks.

### Investigational endpoint

The following tests are to be carried out at the discretion of each investigator according to the availability of the medical devices for the measurement at the screening point and visit 3.

1. Pulse wave velocity

Measurement of the left and right brachial–ankle pulse wave velocity.

2. Central blood pressure

Waveforms directly to be measured from the carotid artery or by applying pressure to the radial artery using a sensor will be obtained using a general transfer formula.

3. 24-h ambulatory BP monitoring

The BP cuff will be wrapped around the upper arm and connected to a portable monitor for monitoring the BP for 24 h. The BP will be measured every 15 min during the day and every 30 min during the night, and the measurement interval can be defined differently.

4. Body fluid composition analysis using InBody720 (body water, edema index)

It will be automatically calculated and confirmed by the measuring equipment.

### Sample size estimation

We have referred to two studies for sample size estimation. One study is regarding a combination therapy with chlorthalidone or valsartan as an add-on drug to diltiazem [19], and the other one compared amlodipine with hydrochlorothiazide versus telmisartan [20]. We have also referred to the Korea Ministry of Food and Drug Safety review data on amlodipine/telmisartan combination [21]. With these references, we calculated the number of patients by assuming the mean change in DBP will be equal in the intervention and control arms and the standard deviation of mean DBP change as 7.2 mmHg in the test group. The alpha error is estimated as 0.05 (one-tailored) and beta error as 0.2. The non-inferiority margin for DBP change in the clinical setting of hypertension is stated as 3 in the Guideline on Assessment by the Ministry of Food and Drug Safety [21]; therefore, we estimated the sample size as 72 and the final size as 85 in each group considering 15% patients will be lost to follow-up.

### Statistical analysis

To identify inter-group difference in the demographic variables and the baseline information at the time point prior to the study initiation, continuous data will be compared by the two-sample *t* test or Wilcoxon’s rank sum test and categorical data by the chi-square test or Fisher’s exact test.

To verify that the mean DBP variation in the S-amlodipine 2.5 mg plus chlorthalidone 25 mg (test) group is non-inferior to that in the S-amlodipine 2.5 mg plus telmisartan 40 mg (control) group, the mean DBP change at week 12 post treatment from the baseline in the two groups will be obtained. If the lower limit of 95% confidence interval is larger than −3 mmHg using the one-tailed *t* test with a significance level of 0.05, the test drug combination would be considered non-inferior to the control drug combination.

Firstly, we will test the normality using the normal probability plot, a quantile–quantile plot (QQ plot), and perform the Kolmogorov–Smirnov test and Shapiro–Wilk test. If normality is not guaranteed, non-parametric tests such as the Mann–Whitney *U* test or Wilcoxon rank sum test will be applied.

Regarding adjustment for covariates, because this is a randomized controlled trial, we believe that the patient characteristics will be well balanced between the groups. However, should there be significant differences between groups, we will adjust for those variables during statistical analysis. ANCOVA will be conducted with the baseline DBP level as a covariate. If the lower limit of the 95% confidence interval for the difference by subtracting the change in the test group from that in the control group is greater than −3 mmHg after ANCOVA, the mean DBP decrease in the test group can be proven to be non-inferior to that in the control group.

### Randomization

Randomization will be performed according to a predesigned block randomization method. The randomization block will be generated by an independent statistician who is unrelated to this study using the SAS randomization program. Randomization will be performed in a 1:1 ratio in a consecutive order.

### Analysis

#### Intention-to-treat analysis set (full analysis set)

Data from all subjects in both groups who are randomly assigned to the primary analysis of efficacy assessment will be subjected to intention-to-treat analysis assuming that the patients have taken the investigational products at least once, and the efficacy assessment will be performed at least once after the time point of baseline evaluation and after the administration of the investigational products.

#### Per-protocol analysis set

In the per-protocol analysis set (PPS) that is subjected to the secondary analysis of efficacy assessment, any of the following subjects who are considered to present major deviations from the clinical study protocol will be excluded from the analysis.

1. Subjects who have not completed the period specified in the study protocol and have withdrawn early.
2. Study subjects whose medication compliance with the investigational products is less than 75%.
3. Study subjects who are determined to present major deviations from the study protocol.
4. Subjects who violate the inclusion/exclusion criteria.

### Safety set

The subjects who are randomly assigned and have taken the investigational products at least once and whose safety-related data are confirmed via telephone or hospital visit by the investigator will be included in the safety set. Efficacy analysis will be conducted on the full analysis set (FAS) and the PPS. Demographic data will be obtained from the FAS, while safety assessment will be performed on the safety set.

In case of missing data at a certain time point or if the subject drops out before the close-out of the clinical study, the last observation carried forward (LOCF) method will be applied to verify the efficacy. The analysis is limited to patients for whom the efficacy assessment has been conducted once from the baseline. The safety analysis will be conducted with the raw data without applying the LOCF method.

## Discussion

Multiple drug therapy ought to be considered by physicians to manage the BP adequately in hypertension patients. Although a number of studies have reported that two drugs or more are needed to control the BP [3, 5, 22], studies on the head-to-head comparison of two-drug combination regimens are scarce. This study compares two regimens of combination therapy in a head-to-head manner to demonstrate the non-inferiority of CCB + diuretic in BP-lowering efficacy and safety compared to CCB + ARB as a combination therapy.

CCBs are one of the most widely used antihypertensive drugs in the world, including Korea, mainly due to their reliable BP-lowering efficacy and low adverse event rate as well as good clinical outcomes [22]. The selection of another medication in addition to CCBs is of importance when the BP is not controlled with CCB monotherapy. ACEIs, ARBs, and diuretics may be combined with CCBs. However, studies comparing the efficacy and safety of these add-on drugs in patients with an uncontrolled BP are lacking. We compare chlorthalidone (diuretic) as the active drug versus telmisartan (ARB) as the comparator after combining with S-amlodipine (CCB). The reason for adding chlorthalidone as the diuretic is its better clinical and preclinical BP-lowering effect, fewer adverse effects, and better clinical outcomes than hydrochlorothiazide. Although little evidence exists on the superiority of chlorthalidone over telmisartan as an add-on to CCB monotherapy, we can assume that chlorthalidone might be more effective in volume-dependent and salt-sensitive hypertension patients than telmisartan; moreover, chlorthalidone is less expensive. Importantly, the lack of evidence regarding the comparison justifies the purpose of this clinical trial.

S-amlodipine, produced by Hanlim Pharmaceutical Co., Ltd., in Korea, is a chiral drug, which was developed because the biological activities of R-amlodipine and S-amlodipine are not the same. Amlodipine is a racemic mixture of S-amlodipine and R-amlodipine in a ratio of 1:1, and S-amlodipine, which acts as a CCB, could be separated by optical methods. S-amlodipine reduces the dose and adverse effects of the racemic mixture of amlodipine. Therefore, the combination of S-amlodipine and chlorthalidone is a good option for controlling the BP in patients unresponsive to CCB monotherapy. Amlodipine plus telmisartan is a good comparator regimen owing to its similarity with the amlodipine plus benazepril combination, the efficacy and safety of which have been reported in the ACCOMPLISH study [5]. Additionally, the BP-lowering effects of both drugs of the combination have been clinically validated [23–25]. Although we propose to investigate the change in BP within a short period, instead of long-term clinical outcomes, we are certain that the results of our study will clarify existing knowledge on the efficacy and safety of CCB + diuretic and will provide clinicians with another option for treating hypertension in patients who do not respond adequately to treatment with a single agent.

Other ancillary parameters, such as pulse wave velocity, central blood pressure, 24-h ambulatory BP monitoring, and body fluid composition analysis, in some patients will give additional insight into the change in aortic stiffness, BP control throughout the day, and effect on edema, which is the major adverse effect of CCBs. If a meaningful difference is observed between the two combinations, it will also allow physicians to better choose drugs in combination, especially for patients experiencing adverse effects of the currently used therapy and in special clinical situations, such as heart failure.

Whether the study results are positive or not, this study is valuable because it is the first comparison between S-amlodipine + chlorthalidone and S-amlodipine + telmisartan and will facilitate the selection of the most appropriate combination regimen for treating patients not responding to single antihypertensive therapy. This study comparing S-amlodipine + chlorthalidone versus S-amlodipine + telmisartan in hypertension patients whose BP is not controlled with amlodipine monotherapy will help clinicians to select the add-on drug for better controlling the BP in hypertension patients.

## Trial status

The trial (ClinicalTrials.gov, identifier number: NCT03226340) is currently ongoing and is in the recruitment phase. Recruitment began on 2 December 2015 and is expected to finish in 2018.

## Additional file

Additional file 1: SPIRIT 2013 checklist: recommended items to address in a clinical trial protocol and related documents. (DOC 124 kb)

## Abbreviations

ARB: Angiotensin receptor blocker
BP: Blood pressure
CCB: Calcium channel blocker
DBP: Diastolic blood pressure
ESC: European Society of Cardiology
FAS: Full analysis set
LOCF: Last observation carried forward
PPS: Per-protocol analysis set
SBP: Systolic blood pressure
